# Personality risk factors in assessing the reliability of the performance of operating personnel

**DOI:** 10.1192/j.eurpsy.2021.1187

**Published:** 2021-08-13

**Authors:** T. Zlokazova, A. Kachina, A. Kuznetsova

**Affiliations:** Faculty Of Psychology, Lomonosov Moscow state University, Moscow, Russian Federation

**Keywords:** human factor error, performance, personality traits, operator’s reliability

## Abstract

**Introduction:**

The development of complex human-machine systems has led to greater demands on operators’ skills, and has increased the importance of human error (Pribytkova et al, 2012; Vondráčková et al, 2017; Jian Ai Yeow, 2014). For this reason performance reliability, defined as operators’ capacity to conduct essential work processes in a high-quality and timely manner (Bodrov, Orlov, 1998) has become a topical subject.

**Objectives:**

This study concerns an investigation of subjective predictors of operators’ reliability, namely personality risk factors (supported by the RFBR #19-013-00799).

**Methods:**

Subjects: 67 operators and 69 engineers at a hydro-power station. Personality traits were assessed using Sobchik’s verbatim Russian translation of the MMPI (Sobchik, 1990). Performance reliability was assessed using simple and complex sensorimotor reaction tests as standard procedures for the pre-shift assessment of operators.

**Results:**

In the operators’ group significant correlations (Spearman’s test) were found between the level of quality of complex sensorimotor reactions and the level of such personal traits as impulsiveness and individualism: a higher manifestation of these traits was associated with a higher level of mistakes in conducting the pre-shift psychophysiological test (p<0,05). With the engineers there was a significant link between the higher speed of simple sensorimotor reactions and higher optimism scores.

**Conclusions:**

The results suggest that a tendency to behave spontaneously, and orientation to one’s own needs, could be risk factors in terms of operator reliability. They also reveal the specifics of reliability predictors in different professions at the power plant.

